# High *MTHFR* promoter methylation levels in men confer protection against ischemic stroke

**DOI:** 10.17305/bjbms.2020.4636

**Published:** 2020-11

**Authors:** Shan Xu, Qianping Shi, Bo Li, Liyuan Han, Guodong Xu, Xiaolin Peng, Hongen Chen, Shuhong Dai, Wancheng Ma, Changyi Wang, Jianping Ma

**Affiliations:** 1 Nanshan Center for Chronic Disease Control, Shenzhen, China; 2 Department of Preventive Medicine, Shantou University Medical College, Shantou, Guangdong, China; 3 Department of Preventive Medicine, School of Medicine, Ningbo University, Ningbo, China; 4 Medical Record Statistics Room, Ningbo Medical Center Lihuili Hospital, Ningbo, China; 5 Luohu Center for Chronic Disease Control, Shenzhen, China

**Keywords:** Ischemic stroke, MTHFR, methylation, Hcy, hypertension

## Abstract

The *MTHFR* gene encodes methylenetetrahydrofolate reductase required for the metabolism of homocysteine (Hcy) – a previously reported independent risk factor for ischemic stroke (IS). In this study, we first aimed to clarify the association between DNA methylation levels in the *MTHFR* promoter and the risk of IS, followed by the analysis of potential interactions between environmental factors and DNA methylation levels that affect IS risk. We recruited 164 patients with hypertension and IS (case group) and 345 age-matched and sex-matched patients with hypertension only (control group). Demographic and clinical information was obtained using questionnaires, and blood samples were collected for biochemical analyses. Fluorescence quantitative methylation-specific PCR (qMSP) was used to detect *MTHFR* promoter methylation levels. A logistic regression analysis was performed to determine the relationship between environmental factors, *MTHFR* promoter methylation levels, and IS risk. We finally generated a receiver operating characteristic curve to determine whether *MTHFR* promoter methylation levels can predict IS. The mean *MTHFR* methylation levels in the case group (8.10 ± 6.14) were significantly lower than those in the control group (17.44 ± 3.16; *p <* 0.05). *MTHFR* promoter methylation levels were also lower in patients with plasma Hcy levels ≥15 μmol/L (10.65 ± 4.05) than in those with Hcy levels <15 μmol/L (16.74 ± 4.26, *p <* 0.001). Finally, we found that *MTHFR* hypermethylation is a protective factor for IS, particular in men (OR in men: 0.07; 95% CI: 0.02–0.16; *p* < 0.001). Further, sex and *MTHFR* promoter methylation levels exhibited a preliminary interaction effect on IS risk. These results indicate that *MTHFR* promoter methylation status might have diagnostic value in IS.

## INTRODUCTION

Stroke is the second leading cause of death and long-term disability worldwide [1,2]. Ischemic stroke (IS) is the most common stroke subtype and accounts for an estimated 63–84% of all stroke cases [3]. IS is a multi-pathogenic disease driven by an interaction between environmental and genetic factors [4]. DNA methylation is an epigenetic, hereditary and reversible process, in which methyl groups are added to CpG islands [5]. This addition affects protein binding by altering the spatial configuration of DNA sequences [6]. DNA methylation constitutes an epigenetic response to environmental factors and is thus considered to function as a molecular bridge between the environment and gene and protein expression [7]. The differential DNA methylation that occurs under different environmental conditions and the subsequent impact on chromatin structure has broad effects on gene expression [8]. Such epigenetic changes have an important role in mediating disease susceptibility [9]. Indeed, studies have shown that abnormal DNA methylation is associated with atherosclerosis [10], hypertension [11], coronary heart disease [12], and stroke [13] risk.

Our previous prospective cohort study found a positive association between hyperhomocysteinemia (HHcy) and IS in a cohort of 5488 patients with hypertension [14,15]. Another study confirmed that patients with hypertension and comorbid HHcy had a >12-fold increased risk of stroke [16]. Homocysteine (Hcy) is a sulfur-containing, non-proteinogenic amino acid that occurs naturally in the plasma [17]. Methylenetetrahydrofolate reductase (MTHFR) is an essential enzyme involved in Hcy metabolism. Genetic mutation of *MTHFR* can cause Hcy deficiency, while a decrease in its activity can lead to increased plasma Hcy levels and HHcy [18,19]. A polymorphism in *MTHFR* is strongly associated with IS [20,21]. We thus aimed to investigate whether *MTHFR* promoter methylation is associated with IS.

## MATERIALS AND METHODS

### Study design and participant recruitment

We recruited 164 patients with both hypertension and IS (case group) and 345 age-matched and sex-matched patients with hypertension only (control group), all from the Nanshan District Community Health Service Center in Shenzhen, China. A diagnosis of IS was confirmed by examination of medical records including reports of symptoms and examination results. The examinations included computed tomography (CT), magnetic resonance imaging (MRI), cerebral angiography, and transcranial Doppler ultrasound, in agreement with the World Health Organization (WHO) criteria, and were conducted by two cerebrovascular experts. Events were also diagnosed as IS if the scan did not visualize an infarction or hemorrhage, but the patient had symptoms that met the WHO criteria for IS.

The inclusion criteria were as follows: 1) patients who were aged ≥20 years and exhibited hypertension, 2) patients who had resided in Shenzhen for >6 months and could ensure their availability for follow-up analyses for at least 3 years, and 3) patients whose health and hypertension records were established in community health service centers. Exclusion criteria were as follows: 1) patients with secondary hypertension, cancer, severe liver, or kidney disease; 2) patients who were pregnant; 3) patients who were taking folic acid or vitamin B6 or B12; and 4) patients who were hypertensive and had a history of stroke or coronary heart disease. A diagnosis of IS was confirmed based on the results of pre-admission symptoms, CT, cranial MRI, cerebral angiography, and transcranial Doppler ultrasound images. All of the patients were informed of the study aims and the analyses that would be performed, and they provided written informed consent before the study started. This study was approved by the Ethics Committee of Nanshan Chronic Disease Prevention Center (approval ID: 1120170008).

### Blood sampling and DNA extraction

CpG islands in the *MTHFR* promoter region were located using the UCSC genome browser. Specific primers to amplify the region by quantitative methylation-specific PCR (qMSP) were then designed using PyroMark Assay Design Software 2.0 (Table 1). After 12 hours of fasting, 5 mL venous blood was collected by venipuncture from each of the study participants into an ethylenediaminetetraacetic acid (EDTA) anticoagulant tube and immediately transported on ice to the laboratory for follow-up analyses or preservation at -80°C. DNA from each blood sample was extracted using a Lab-Aid 820 Nucleic Acid Extraction System (Xiamen Zhishan Biotechnology Co., Ltd.). The DNA concentrations and purities were measured on a NanoDrop 1000 spectrophotometer (Thermo Fisher Scientific Corporation, USA).

**TABLE 1:**
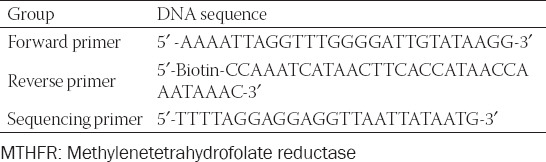
Pyrophosphate sequencing primers for CpG islands in the promoter region of *MTHFR* gene

### Methylation analysis

DNA bisulfate conversion was achieved using an EZ DNA Methylation-Gold Kit (Zymo Research Corporation, USA), according to the manufacturer’s instructions. The *MTHFR* promoter methylation status was then detected by qMSP [22]. The qMSP reaction consisted of 1.5 μL bisulfate-converted DNA, 0.5 μL forward primer, 0.5 μL reverse primer, 10 μL Zymo TaqTM PreMix, and 7.5 μL DNase-free and RNase-free water. The PCR cycling conditions were as follows: pre-denaturation at 95°C for 10 minutes, denaturation at 95°C for 20 seconds, 45 cycles of annealing at 56–58°C for 45 seconds, and an extension at 72°C for 20 seconds, final extension at 40°C for 5 minutes, and preservation at 4°C. After PCR amplification, the methylation level was detected on a Qsep100 DNA Analyzer (BiOptic Inc., China). The percentage of methylated reference (PMR) was used to quantify the methylation level [22]. The PMR value was obtained as previously described [22] by converting the PCR cycle threshold (Ct) value, as follows: PMR = 2-(Ct sample - Ct internal reference) × 100%.

### Questionnaire-based data collection and physical measurements

Questionnaires and physical measurements were used to collect information on various environmental factors. The questionnaire collected information on the cohort demographics (including name, sex, birth date, nationality, occupation, education level, and marital status), health-related behaviors, dietary and emotional status, history of illness, and history of drug use. A questionnaire for dietary frequency was used to collect information on the consumption of foods rich in folic acid and vitamins, such as coarse grains, vegetables, and fruits. The Zung self-rating depression scale (SDS) [23] was used to evaluate the participants’ emotional status. Here, emotional status is based on the answers given to 20 questions; each question is scored on a scale from 1 to 4. The maximum total score is 100: the lower the score, the better the emotional state. The upper limit of a normal score is 49 and participants with an SDS standard score ≥50 were considered to exhibit depressive symptoms. The physical measurements included height, weight, waist circumference, hip circumference, and blood pressure.

### Biochemical analyses

The blood samples were analyzed for fasting blood glucose (Glu), total cholesterol (TC), low-density lipoprotein (LDL) cholesterol, triglyceride (TG), uric acid, creatinine, and plasma Hcy levels. An automatic biochemical analyzer (HITACHI 7080, Japan) was used to detect these blood biochemical indexes.

### Statistical analysis

The qualitative data are presented as percentages (%) and a Chi-square test was used for statistical testing. The quantitative data are presented as means ± standard deviation, and a *t*-test was used to make comparisons between the groups. The methylation level of the *MTHFR* promoter region was divided into four quartiles (Q1, Q2, Q3, and Q4) and then analyzed by multivariate logistic regression analysis. An odds ratio (OR) with 95% confidence intervals (CIs) was used to express the correlation between the influencing factors and *MTHFR* methylation levels.

Logistic regression was used to construct three models: M1, M2, and M3. The fourth model, M0, did not correct for other factors. M1 corrected for age and gender. M2 corrected for smoking, body mass index (BMI), waist-hip ratio (WHR), depression, sleep, and oil and salt intake on the basis of M1. On the basis of M2, M3 corrected for factors such as systolic blood pressure (SBP), fasting Glu, TC, TG, LDL, and Hcy levels. Based on M3, the area under the receiver operating characteristic (ROC) curve was evaluated and used to determine the effect of the logistic regression model.

All of the statistical analyses were two-sided and a *p* value <0.05 was considered statistically significant. All of the statistical analyses were performed using IBM SPSS Statistics for Windows, Version 20.0. (IBM Corp., Armonk, NY, USA).

## RESULTS

### Hcy and *MTHFR* promoter methylation levels vary between cases and controls

We found significant differences in the *MTHFR* promoter methylation levels and Hcy status between the two groups. Specifically, the Hcy levels were 16.79 ± 1.62 μmol/L in the case group and 15.06 ± 1.45 μmol/L in the control group (*p* = 0.011). The *MTHFR* promoter methylation levels were also significantly lower in the case group (8.1 ± 6.14) than in the control group (17.44 ± 3.16, *p* < 0.001; Table 2).

**TABLE 2:**
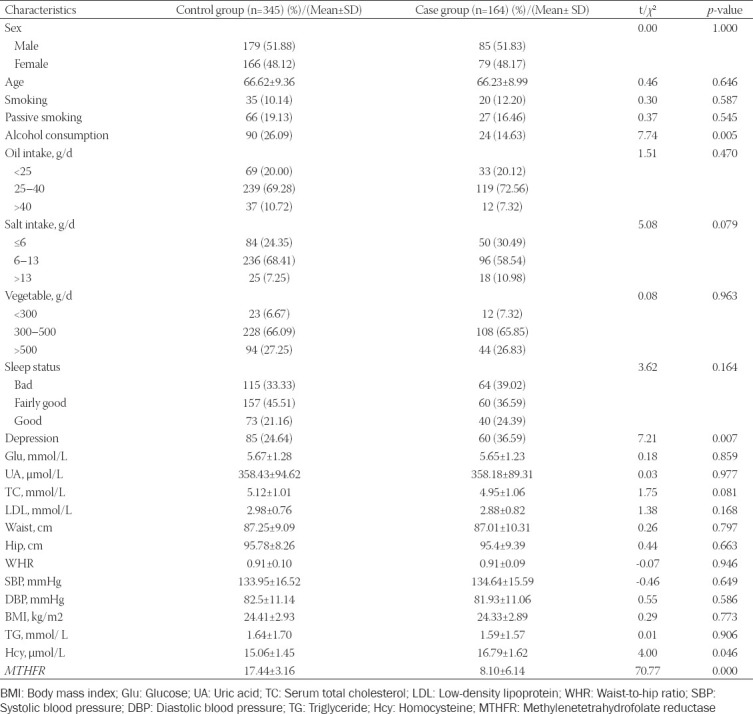
Characteristics of 164 cases and 345 controls included in the study

### *MTHFR* promoter methylation levels vary with Hcy status

We found a significant difference in the *MTHFR* promoter methylation levels between the two groups in terms of the Hcy status (*p* < 0.001, Table 3). Specifically, the *MTHFR* promoter methylation levels were 16.74 ± 4.26 in patients with an Hcy level <15 μmol/L (group 1) and 10.65 ± 4.05 in patients with an Hcy level >15 μmol/L (group 2).

**TABLE 3:**
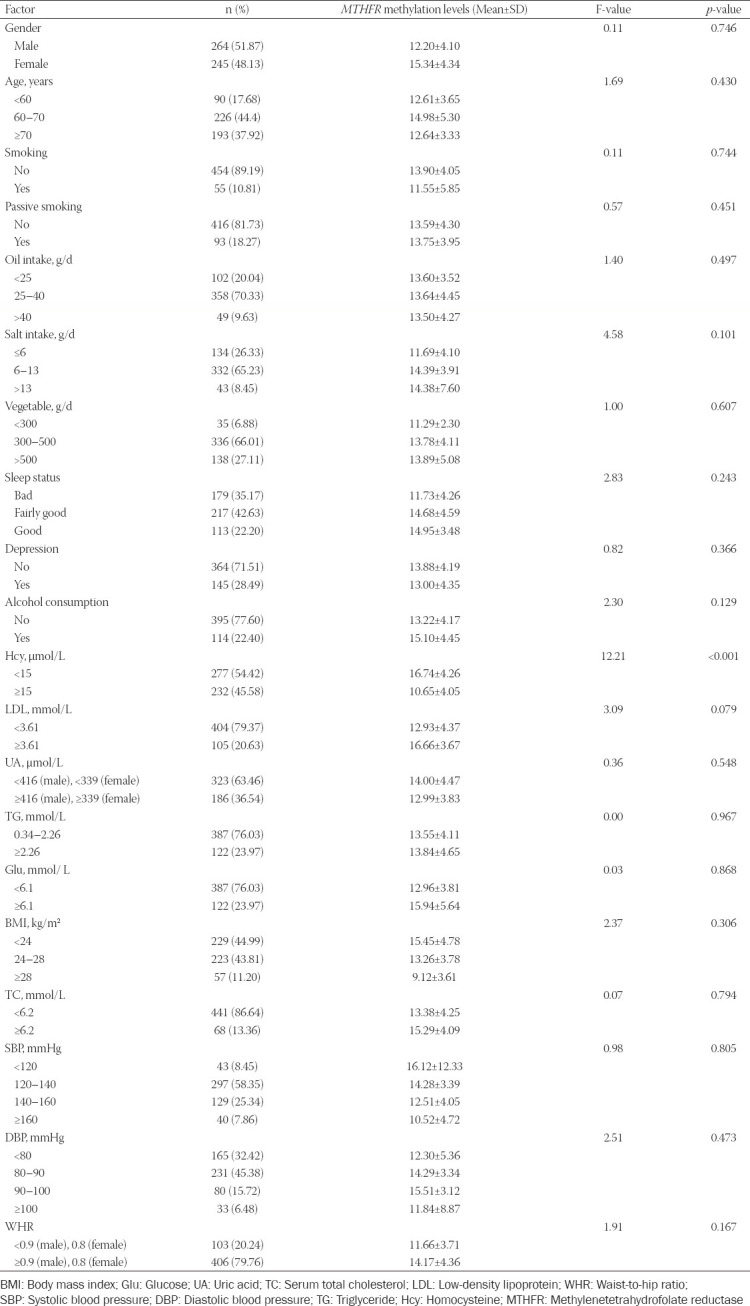
*MTHFR* methylation levels in different environmental factors

### High *MTHFR* promoter methylation levels protect against IS

Univariate analysis showed that depression, alcohol consumption, and *MTHFR* promoter methylation levels influenced IS risk (*p* ≤ 0.01, Table 4). Multivariate analysis confirmed that depression, alcohol consumption, and *MTHFR* promoter methylation levels influenced IS risk (all *p* ≤ 0.01, Table 4).

**TABLE 4:**
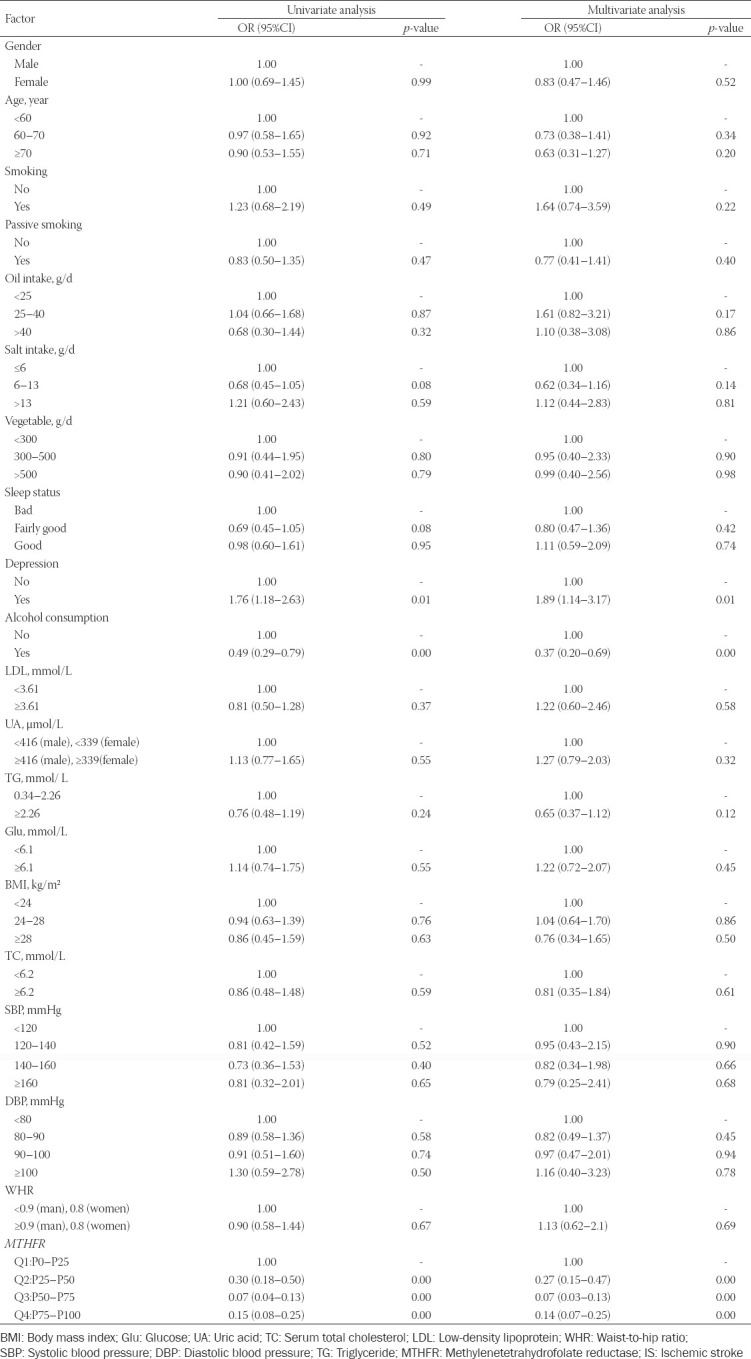
Regression analysis of the association between environmental factors and IS

### High *MTHFR* promoter methylation levels confer protection against IS in men

We next constructed four models to analyze the relationship between IS and *MTHFR* promoter methylation levels by quartiles. After correcting for age, sex, smoking, BMI, WHR, depression, sleep, oil and salt intake, SBP, Glu, TC, TG, LDL and Hcy levels, we found that for the highest quartile of *MTHFR* promoter methylation level (Q4), the total OR (95% CI) of IS was 0.13 (0.07–0.24). The OR (95% CI) in men was 0.07 (0.02–0.16), which was lower than that in women (0.23 [0.09–0.54]). After correcting for the related factors in M2, the total OR (95% CI) was 0.14 (0.08–0.25) when compared with the lowest quartile (Q1). The protective effect in men (0.07 [0.03–0.16]) remained stronger than that in women after correction. After correcting for M3, the *MTHFR* promoter methylation levels in men had a statistically significant *p*-trend with IS (all *p* < 0.01; Table 5).

**TABLE 5:**
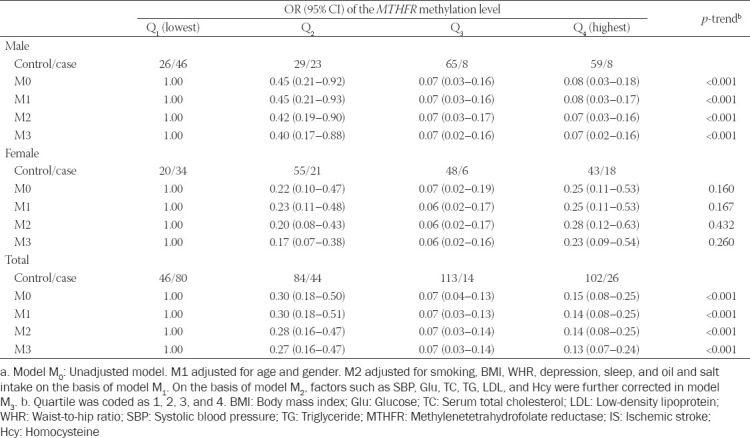
The relationship between *MTHFR* methylation level and ischemic stroke^a^

### Interaction between sex and *MTHFR* promoter methylation levels affects IS risk

The interaction between sex and *MTHFR* promoter methylation levels was found to significantly affect IS risk (*p* = 0.011; Table 6). We found no interaction between age, depression, sleep, Hcy, Glu, and *MTHFR* promoter methylation levels in relation to IS risk (all *p* > 0.05).

**TABLE 6:**
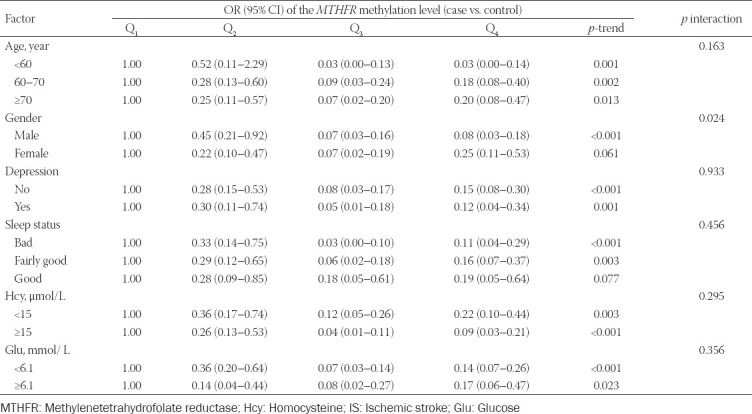
The effects of the interaction between *MTHFR* methylation level and environmental factors on IS

### *MTHFR* promoter methylation levels predict IS

**FIGURE 1:**
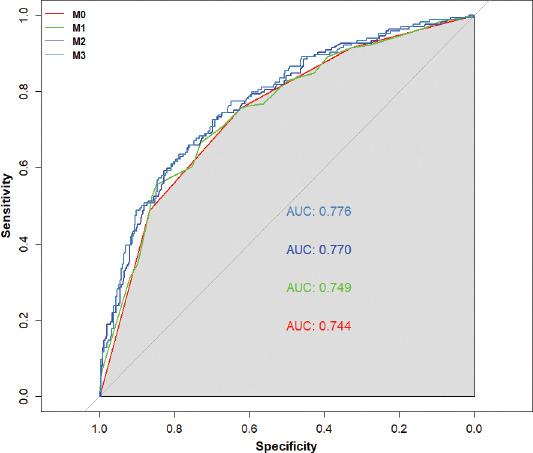
A receiver operating characteristic curve showing that methylenetetrahydrofolate reductase (*MTHFR*) promoter methylation levels could predict ischemic stroke.

To evaluate the predictive value of *MTHFR* promoter methylation levels on IS risk, we calculated the area under the ROC curve. We found that *MTHFR* promoter methylation levels could predict IS with ORs of 0.744 (95% CI: 0.702–0.786) at M0, 0.749 (95% CI: 0.712–0.797) at M1, 0.770 (95% CI: 0.749–0.832) at M2, and 0.776 (95% CI: 0.762–0.844) at M3, respectively (Figure 1).

## DISCUSSION

In this study, we analyzed the correlation between *MTHFR* promoter methylation levels and IS risk, and the potential interactions between *MTHFR* promoter methylation levels and environmental factors. We recruited 164 patients with both hypertension and IS (cases) and 345 patients with hypertension without IS (controls). We found that high levels of *MTHFR* promoter methylation reduced the risk of IS in patients with hypertension by 86% compared with those with low levels of *MTHFR* promoter methylation, after adjusting for potential confounders. Increased *MTHFR* promoter methylation levels had a stronger protective effect on IS risk in men than in women.

IS is a complex neurological disease caused by both genetic and environmental factors [24]. DNA methylation profiles have been associated with many of the pathological changes that accompany aging. DNA methylation plays an important role in regulating gene expression and has the potential to modulate IS risk [25]. A previous study that performed a luminometric methylation assay found no correlation between total DNA methylation levels and IS [26]. Another study found that total DNA hypomethylation increased the risk of IS [27]. Here, we found that high levels of *MTHFR* promoter methylation reduced IS risk in patients with hypertension.

*MTHFR* encodes one of the enzymes required for the metabolism of Hcy – a previously reported independent risk factor for IS [24]. Folic acid also antagonizes HHcy formation during Hcy metabolism [28]. Nagele et al. [22] found that extensive folic acid fortification in the population could significantly reduce Hcy plasma levels and possibly reduce the role of the *MTHFR* C677T polymorphism in increasing plasma Hcy levels. Wei et al. [29] found that *MTHFR* promoter methylation profiles at CpG islet A were associated with the serum levels of folate and vitamin B12 – the coenzymes involved in one-carbon metabolism, and that one-carbon metabolism has an important role in modulating DNA methylation. Vitamin B12 or folate can reduce S-adenosylmethionine bioavailability, which hinders genome-wide methylation and, specifically, lysine 4 histone H3 trimethylation (H3K4me3). Moreover, a low level of H3K4me3 might affect *MTHFR* transcriptional activity [29].

We found that the protective effect of *MTHFR* hypermethylation on IS was more pronounced in men. Sebag et al. [30] found that in both male and female mice, DNA methylation patterns were associated with the stability of DNA sequences and that life events had notable effects on DNA methylation in somatic cells. A possible explanation for this phenomenon is the effects of sex hormones on DNA methylation. They also found differential CpG methylation at the *CSQ2* promoter between male and female mice and concluded that sex hormones are necessary to maintain the DNA methylation patterns at this locus. However, the extent to which DNA methylation at specific sites in humans is affected by sex, genetic and environmental factors needs further study. We found no difference between the ROC curves that were adjusted or not for potential confounders (age, sex, etc.). This finding implies that *MTHFR* promoter methylation has a consistent, significant effect on IS risk.

A strength of our study is its analysis of a cohort of participants that share a similar genetic background and have established health records in community health service centers. We collected the data conforming to rigid quality controls, using a questionnaire that was verified to be reliable. Some limitations to this study should, however, be noted. The inherent nature of this case–control, retrospective study means that it cannot provide direct causal associations. In addition, recall bias might have affected the results, as the data on lifestyle were self-reported. The frequency of vegetable intake was also self-reported; as such, we could not calculate the specific consumption of B6, B12 and folic acid, which might be potential confounders.

## CONCLUSION

We showed that high levels of *MTHFR* promoter methylation are protective against IS in patients with hypertension. Only sex and *MTHFR* promoter methylation levels showed interaction effects on IS. These data support the potential application of *MTHFR* promoter methylation status as a predictive biomarker for IS, pending future confirmatory studies.
